# Higher incidence of acute kidney injury in patients treated with piperacillin/tazobactam than in patients treated with cefepime: a single-center retrospective cohort study

**DOI:** 10.1186/s40780-019-0142-6

**Published:** 2019-06-12

**Authors:** Shota Kadomura, Yoh Takekuma, Yuki Sato, Masato Sumi, Kotaro Kawamoto, Tatsuya Itoh, Mitsuru Sugawara

**Affiliations:** 1 Department of Pharmacy, Japan Community Healthcare Organization Sapporo Hokushin Hospital, 6-2-1, Atsubetsuchuo 2-jo, Atsubetsu-Ku, Sapporo, 004-8618 Japan; 2 Graduate School of Life Science, Hokkaido University, Kita-12-jo, Nishi-6-Chome, Kita-Ku, Sapporo, 060-0812 Japan; 3 Faculty of Pharmaceutical Sciences, Hokkaido University, Kita-12-jo, Nishi-6-Chome, Kita-Ku, Sapporo, 060-0812 Japan

**Keywords:** acute kidney injury, nephrotoxicity, piperacillin/tazobactam, cefepime, beta-lactams

## Abstract

**Background:**

Piperacillin/tazobactam (PIPC/TAZ) and cefepime (CFPM) are commonly used for the treatment of nosocomial and healthcare-associated infections. Recent reports have suggested that the incidence of acute kidney injury (AKI) in patients treated with a combination of vancomycin (VCM) and PIPC/TAZ is higher than that in patients treated with CFPM. However, there have been few reports on a comparison of the incidences of AKI in patients treated with PIPC/TAZ monotherapy and patients treated with CFPM. In this study, we investigated whether the incidence of AKI in patients treated with PIPC/TAZ is higher than that in patients treated with CFPM.

**Methods:**

This study was a single-center retrospective observational study. Patients who died during the therapeutic period, patients younger than 18 years of age, and patients undergoing hemodialysis were excluded. Primary outcomes were the incidence of AKI and the AKIN stages defined by the Acute Kidney Injury Network. Secondary outcomes were discontinuation and/or change of antibiotics and initiation of dialysis due to AKI. We also investigated the time to onset and the risk factors of AKI in this population.

**Results:**

There were 163 patients in the PIPC/TAZ group and 103 patients in the CFPM group. The incidence of AKI in patients treated with PIPC/TAZ (8.6%) was significantly higher than that in patients treated with CFPM (0.9%) (odds ratio (OR), 9.53; 95% confidence interval (CI), 1.41–408; p= 0.011). AKI severity was mostly stage 1 in both groups. There was no discontinuation and/or changes of antibiotics and there was no initiation of dialysis in either group. The onset of AKI in the PIPC/TAZ group (median period of 4 days) was earlier than that in the CFPM group. PIPC/TAZ was determined to be an independent risk factor of AKI in multivariate analysis (adjusted OR, 9.56; 95% CI, 1.21–75.3; p = 0.032).

**Conclusions:**

This study showed that the incidence of AKI in patients who received PIPC/TAZ was higher than that in patients who received CFPM. Furthermore, the onset of AKI was earlier in patients who received PIPC/TAZ than in patients who received CFPM. PIPC/TAZ was an independent risk factor of AKI in this study population.

## Introduction

Piperacillin/tazobactam (PIPC/TAZ) is a combination medication containing an antipseudomonal penicillin and a beta-lactamase inhibitor and it is widely used for treatment of nosocomial and healthcare-associated infections such as pneumonia, complicated urinary tract infection, sepsis and febrile neutropenia. Cefepime (CFPM) is an antipseudomonal cephalosporin and is frequently used for treatment of same infections that are treated with PIPCTAZ. Both types of antibiotics are beta-lactam antibacterial agents and are often administered with vancomycin (VCM) to target methicillin-resistant gram-positive organisms in cases of severe infection such as catheter-related bloodstream infection.

Several groups have reported higher rates of acute kidney injury (AKI) in patients treated with the combination of PIPC/TAZ and VCM than in patients treated with VCM alone [1] or CFPM plus VCM [2]. However, there are no comparative data for the incidence of AKI in patients treated with PIPC/TAZ monotherapy and patients treated with CFPM. Karino et al. reported that the incidence of PIPC/TAZ-induced nephrotoxicity in elderly patients was 18.2% (4/22) [3], although the incidence of AKI in Japanese patients administered PIPC/TAZ was reported to be 0.4% (2/486) from post-marketing surveillance data. Accordingly, the actual incidence of AKI in patients who have received PIPC/TAZ may be higher. In contrast, the risk of AKI in patients receiving CFPM is presumed to be very low because there has been only one reported case of interstitial nephritis in patients treated with CFPM [4].

The aim of this study was to determine the incidences and times of onset of AKI in patients treated with PIPC/TAZ and patients treated with CFPM and to identify the risk factors of AKI.

## Methods

This study was a single-center retrospective cohort study conducted in a 276-bed secondary-care hospital in Japan. Patients were recruited between January 1, 2012 and July 30, 2016 for the PIPC/TAZ group and between January 1, 2009 and July 30, 2016 for the CFPM group. Inclusion criteria were hospitalized patients who received either of the antimicrobial agents for 2 days or longer and in whom serum creatinine (SCr) and blood urea nitrogen were measured before and after administration. Patients who died during the therapeutic period, patients under 18 years of age, patients who also received vancomycin and patients who underwent chronic hemodialysis were excluded.

### Data collection

Data obtained from medical records were reviewed and analyzed. We collected data for age, sex, laboratory measurements, estimated glomerular filtration rate (eGFR) (calculated by the Japan Association of Chronic Kidney Disease Initiatives equation using serum creatinine [5]), daily doses of and periods of treatment with PIPC/TAZ and CFPM, infectious diagnosis, comorbidities including hypertension, heart failure, diabetes, malignancy, benign prostatic hyperplasia, chronic kidney diseases (defined as eGFR of less than 60 mL/min/1.73m^2^ using the SCr levels before PIPC/TAZ or CFPM administration), administration of nephrotoxic agents including systemic non-steroidal anti-inflammatory drugs, angiotensin II-converting enzyme inhibitors and/or angiotensin receptor antagonists, diuretics, systemically administered calcineurin inhibitors, catecholamines (norepinephrine and/or dopamine), intravenous aminoglycosides, systemically administered acyclovir, intravenous amphotericin-B, cisplatin and contrast media (within 72 hours before the therapeutic period or during PIPC/TAZ or CFPM administration).

### Outcomes

The primary outcomes assessed in this study were the incidence of AKI and the stage of AKI defined by the Acute Kidney Injury Network (AKIN) criteria [6]. AKI was defined as an elevation in SCr by ≥0.3 mg/dL (within 48 hours) or ≥50% from the pre-treatment most recent data during the therapeutic period. AKIN criteria were categorized into 3 stages of AKI: an absolute increase in SCr level of ≥0.3 mg/dL or a 1.5-fold increase was categorized as stage 1, a 2-fold increase in SCr was categorized as stage 2, and an increase in SCr of 3-fold or ≥ 4 mg/dL or initiation of renal replacement therapy was categorized as stage 3. The secondary outcomes were the discontinuation and/or change of antibiotics and initiation of renal replacement therapy during the therapeutic period. The secondary outcomes were discontinuation and/or change of antibiotics and initiation of renal replacement therapy during the therapeutic period.

### Sample size

To detected a 9% difference in the incidence of AKI between in the PIPC/TAZ group and the CFPM group, a sample size of 194 patients (97 cases in each group) was estimated to achieve a statistical power of 80% based on the estimates of a 10% risk of AKI in the PIPC/TAZ group and a 1% risk of AKI in the CFPM group. The incidence of AKI in patients who received PIPC/TAZ was estimated from a previous report [7]. The type I error (α) is 0.05.

### Times to onset of AKI in patients who received PIPC/TAZ and patients who received CFPM

We investigated the times to onset of AKI after administration of PIPC/TAZ and after administration of CFPM. Kaplan-Meier analysis was performed for the occurrence of AKI in patients who received PIPC/TAZ and patients who received CFPM.

### Analysis of risk factors associated with AKI

In univariate analysis, we extracted statistically significant factors related to AKI in the study population. Additionally, we selected the explanatory variables associated with AKI and analyzed in multivariate analysis.

### Statistical analysis

Descriptive and demographic categorical variables were compared using Fisher’s exact test. Continuous variables were compared using the Mann-Whitney U-test. All p-values were two-sided and a p-value less than 0.05 was considered statistically significant. Multivariate analysis was performed using a logistic regression model. Kaplan-Meier analysis was used to estimate the incidence of AKI, and probabilities in the groups were compared using the log-rank test. All statistical analyses were performed by EZR (Easy R) v1.32 [8].

## Results

### Patient characteristics

**Fig. 1:**
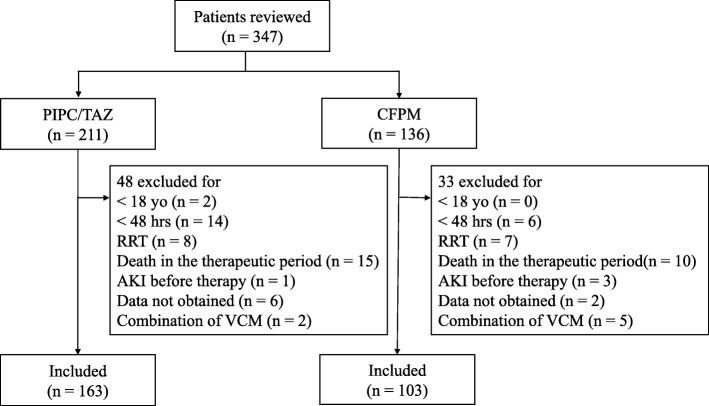
Flow diagram for patient selection. AKI, acute kidney injury; CFPM, cefepime; PIPC/TAZ, piperacillin/tazobactam; RRT, renal replacement therapy; VCM, vancomycin

We enrolled 163 patients in the PIPC/TAZ group and 103 patients in the CFPM group (Figure 1). All of the patients received intermittent infusion (30 minutes ~ 1 hour). Continuous infusion was not performed. Table 1 shows the characteristics of patients in this study. The mean age of the patients was about 70 years in both groups. The proportion of females was higher in the PIPC/TAZ group. The median period of therapy in the PIPC/TAZ group was shorter than that in the CFPM group. In both groups, respiratory tract infection was the most common infection. Abdominal infection and urological infection were frequent in the PIPC/TAZ group, and febrile neutropenia and fever of unknown origin were frequent in the CFPM group. There was no significant difference between comorbidities in the two groups. There was also no difference between the values of eGFR in the two groups. In the PIPC/TAZ group, the proportion of patients who received contrast media was larger and the proportion of patients who received NSAIDs was smaller than those in the CFPM group.

**Table 1:** Patients’ characteristics

Characteristics	PIPC/TAZ (*n* = 163)	CFPM (*n* = 103)	*p*-value
Age (years)			
Median (range)	76.0 (21 – 96)	75.0 (26 – 95)	0.99
Female, *n* (%)	61 (37.4)	24 (23.3)	0.021
Dose frequency per day, *n*			
1, 2, 3, 4	0, 15, 139, 9	6, 90, 7, 0	-
Duration of therapy (days)			
Median (range)	6 (2 – 24)	8 (3 – 26)	< 0.001
Infectious diagnosis, *n* (%)			
Respiratory tract	75 (46)	58 (56)	-
Abdomen	36 (22)	6 (6)	-
Urinary tract	15 (9)	7 (7)	-
Neutropenia	10 (6)	18 (18)	-
Sepsis	8 (5)	4 (4)	-
Fever of unknown origin	8 (5)	7 (7)	-
Skin and soft tissue	5 (3)	1 (1)	-
Catheter-associated BSI	3 (2)	2 (2)	-
Head and neck	3 (2)	0	-
Eye	0	1 (1)	-
Comorbidity, *n* (%)			
Hypertension	84 (52)	46 (45)	0.31
Heart failure	26 (16)	13 (13)	0.48
Diabetes	46 (28)	26 (25)	0.67
Malignancy	49 (30)	44 (42)	0.047
Prostatic hypertrophy	32 (20)	17 (17)	0.63
Chronic kidney disease	68 (42)	39 (38)	0.61
30-day mortality, n (%)	10 (6.1)	9 (8.7)	0.42
Serum creatinine (mg/dL)			
Median (IQR)	0.92 (0.66 – 1.25)	0.84 (0.64 – 1.29)	0.68
eGFR (mL/min/1.73m^2^ )			
Median (IQR)	62.5 (40.2 – 80.8)	64.9 (40.1 – 86.3)	0.35
Concomitant, *n* (%)			
Contrast media	28 (17)	2 (2)	< 0.001
NSAIDs (i.v. or p.o.)	66 (41)	63 (63)	0.0011
ACE-I / ARB	50 (31)	29 (27)	0.68
Diuretics	37 (23)	29 (28)	0.38
Calcineurin inhibitors (p.o.)	1 (0.6)	1 (0.9)	1
Catecholamine	8 (5)	2 (2)	0.32
Aminoglycoside (i.v.)	1 (0.6)	1 (0.9)	1
Acyclovir (p.o.)	1 (0.6)	0	1
Cisplatin	1 (0.6)	0	1

*IQR* Interquartile range, *NSAIDs* Non-steroidal anti-inflammatory drugs, *ACE-I* Angiotensin-converting enzyme inhibitors, *ARB* Angiotensin-II receptor blockers, i.v. Intravenous, p.o.: oral

### Outcomes

**Fig. 2:**
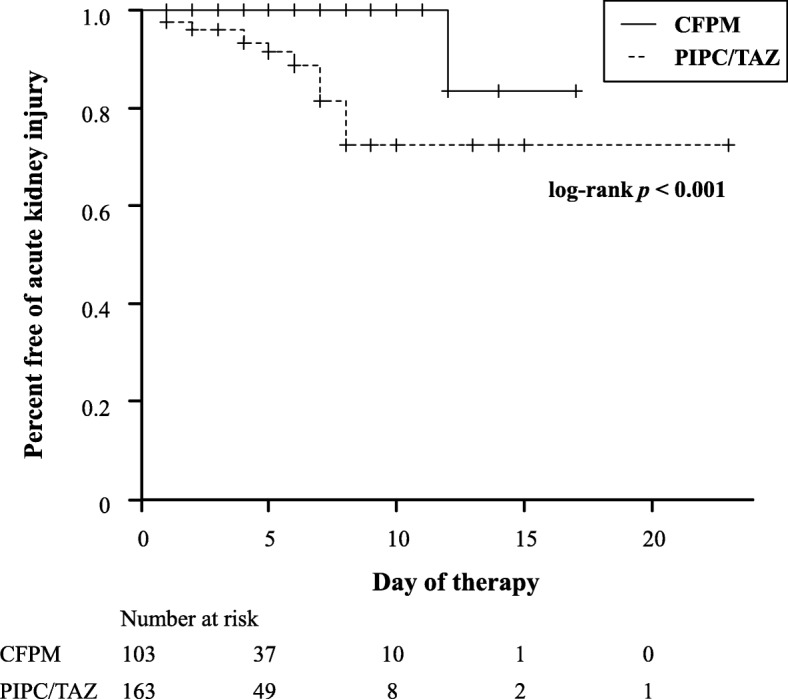
Kaplan-Meier curve of acute kidney injury in each group. The solid line shows the piperacillin/tazobactam (PIPC/TAZ) group and the dashed line shows the cefepime (CFPM) groupsss

The incidence of AKI in patients treated with PIPC/TAZ was more than 9-times higher than that in patients treated with CFPM (Table 2). AKI stage 1 in the AKIN criteria was the most common stage in both groups. Secondary outcomes were not observed in either group. Kaplan-Meier estimates of the incidence of AKI after antimicrobial therapy are shown in Figure 2. There was a significant difference between the two groups (log-rank test, *p* < 0.001).

**Table 2:** Outcomes of nephrotoxicity in patients who received PIPC/TAZ and CFPM

Outcomes	PIPC/TAZ (*n* = 163)	CFPM (*n* = 103)	*p*-value
Acute kidney injury, *n* (%)	14 (8.6)	1 (0.9)	
Odds ratio [95% CI]	9.53 [1.41 – 408]	reference	0.011
AKIN grade			
stage 1, *n*	12	1	
stage 2, *n*	2	0	
stage 3, *n*	0	0	
Discontinuation or change of antibiotics	0	0	-
Initiation of dialysis	0	0	-

*CI* Confidence interval, *AKIN* Acute Kidney Injury Network

### Times to onset of AKI in patients who received PIPC/TAZ and patients who received CFPM

Kaplan-Meier curves in Figure 2 show the onset of AKI after administration of antibiotics. The median time of onset of AKI in the PIPC/TAZ group (4 days, interquartile range (IQR): 2-6) was earlier than that in the CFPM group.

### Analysis of risk factors associated with AKI

The characteristics of patients in whom AKI occurred (AKI group) and patients in whom AKI did not occur (Non-AKI group) are shown in Table 3. Three factors (PIPC/TAZ, CKD, diabetes) were extracted in univariate analysis. Multivariate analysis in the logistic regression model showed that independent risk factors were PIPC/TAZ, CKD and DM (Table 4).

**Table 3:** Characteristics of patients in the AKI group and the non-AKI group

Parameters	AKI group ( *n* = 15)	Non-AKI group ( *n* = 251)	*p*-value
PIPC/TAZ, *n* (%)	14 (93.3)	149 (59.4)	0.011
Age, median (range)	80 (59 – 96)	75 (21 – 95)	0.31
Female, *n* (%)	6 (40.0)	79 (31.5)	0.57
Hypertension, *n* (%)	8 (53.3)	122 (48.6)	0.79
Heart failure, *n* (%)	5 (33.3)	34 (13.5)	0.051
Diabetes, *n* (%)	9 (60.0)	59 (23.5)	0.006
Malignancy, *n* (%)	7 (46.7)	86 (34.3)	0.40
Prostatic hypertrophy, *n* (%)	4 (26.7)	45 (17.9)	0.49
CKD, *n* (%)	12 (80.0)	95 (37.8)	0.0019
Contrast media, *n* (%)	2 (13.3)	27 (10.8)	0.67
NSAIDs, *n* (%)	5 (33.3)	124 (49.4)	0.29
ACE-I / ARB, *n* (%)	6 (40.0)	73 (29.1)	0.39
Diuretics, *n* (%)	7 (46.7)	59 (23.5)	0.062
Calcineurin inhibitors, *n* (%)	0 (0)	2 (0.8)	1
Catecholamine, *n* (%)	2 (13.3)	8 (3.2)	0.10
Aminoglycoside, *n* (%)	0 (0)	2 (0.8)	1
Acyclovir, *n* (%)	0 (0)	1 (0.4)	1
Cisplatin, *n* (%)	0 (0)	1 (0.4)	1

*PIPC/TAZ* Piperacillin/tazobactam, *CKD* Chronic kidney disease, *NSAIDs* Non-steroidal anti-inflammatory drugs, *ACE-I* Angiotensin-converting enzyme inhibitors, *ARB* Angiotensin-II receptor blockers

**Table 4:** Univariate and multivariate analyses (logistic regression analysis)

Parameters	Crude OR (95% CI)	*p*-value	Adjusted OR (95% CI)	*p*-value
PIPC/TAZ	9.53 (1.41 – 408)	0.011	9.56 (1.21 – 75.3)	0.032
CKD	6.52 (1.70 – 36.9)	0.0019	5.06 (1.33 – 19.2)	0.017
Diabetes	4.45 (1.35 – 15.8)	0.006	3.16 (1.02 – 9.78)	0.045

*OR* Odds ratio, *CI* Confidence interval, *PIPC/TAZ* Piperacillin/tazobactam, *CKD* Chronic kidney disease

## Discussion

In this study, we investigated whether the incidence of AKI in patients who received PIPC/TAZ is higher than that in patients who received CFPM. This study is the first study in which the incidences of AKI in patients who received PIPC/TAZ monotherapy and patients who received CFPM were compared. Our results showed that patients who receive PIPC/TAZ have a 9-times higher risk of AKI than do patients who receive CFPM.

A previous study showed that the incidences of AKI in patients who received intermittent infusion and those who received continuous infusion of PIPC/TAZ were 9% and 11%, respectively [7]. The overall incidence of AKI in our study was 8.6% (14/163), which is similar to the results of that previous study. Another report showed an incidence of AKI of 18.4% in Japanese elderly patients who were diagnosed with nursing and healthcare-associated pneumonia and administered PIPC/TAZ [9]. Our results showed a lower incidence of AKI in patients who received PIPC/TAZ. The reason for the difference in results might be the exclusion of patients less than 65 years of age in that study. In contrast, our study suggested that few cases of AKI occur in patients treated with CFPM.

Most of the patients in whom AKI occurred had AKIN stage 1, and discontinuation of administration or initiation of renal replacement therapy was not needed in any of the patients. Rutter and colleagues reported that the incidence of AKI in patients who received PIPC/TAZ monotherapy was 7.8% and that most of the patients who had AKI were classified as “*risk”* and none of the patients were classified as “*loss*” or “*end-stage kidney diseases*” in the RIFLE criteria [10]. Those results are similar to our results showing that AKI associated with PIPC/TAZ was mainly mild dysfunction. In most of the patients in whom AKI occurred, SCr levels were reversible to baseline levels.

The onset of AKI was within 7 days after PIPC/TAZ administration in most of the patients in our study (median period of 4 days). To the best of our knowledge, there have been few reports about the onset of AKI in patients treated with PIPC/TAZ. Morimoto et al. showed in a retrospective observational study that AKI caused by PIPC/TAZ occurred mostly within 7 days in patients with pneumonia [11]. Additionally, Navakelle and colleagues showed that the onset of AKI in patients treated with a combination VCM and PIPC/TAZ (median period of 3 days) was more rapid than that in patients treated with VCM and CFPM (median period of 5 days) [12]. Those results are similar to our results showing that the onset of AKI in the PIPC/TAZ group was earlier than that in the CFPM group. Furthermore, the results suggested that the incidence of AKI in patients treated with the combination of VCM and CFPM was related to VCM trough levels but that the incidence of AKI in patients treated with the combination of VCM and PIPC/TAZ was not associated with VCM trough levels. It is conceivable that nephrotoxicity of PIPC/TAZ develops at an early timing by another mechanism and is not related to the cumulative dose and therapeutic duration. Jensen and colleagues reported that the renal recovery rate in critically ill patients treated with PIPC/TAZ was lower than that in critically ill patients treated with other antibiotics [13]. Their report suggests that PIPC/TAZ monotherapy affected the patients’ renal function. Burgess and colleagues reported that the incidence of VCM-induced nephrotoxicity in hospitalized patients who received VCM in combination with PIPC/TAZ (16.3%) was higher than that in patients who received VCM without PIPC/TAZ (8.08%) [1]. In addition, Gomes and colleagues reported that the incidence of AKI in patients treated with a combination of VCM and PIPC/TAZ (34.8%) was higher than that in patients treated with CFPM (12.5%) [2]. The incidence of AKI in patients treated with CFPM was low in our study. Moreover, it has been reported that only one case of interstitial nephritis occurred due to administration of CFPM [4]. Consequently, it seems that CFPM rarely affects the incidence of AKI in patients who receive a combination of CFPM with VCM, although the incidence of VCM-induced nephrotoxicity has varied [14]. In contrast, the incidence of AKI in patients who received PIPC/TAZ monotherapy was significantly higher than that in patients who received CFPM in our study. Therefore, PIPC/TAZ-induced nephrotoxicity might be related to the higher incidence of AKI in patients who received a combination VCM and PIPC/TAZ than in patients who received VCM alone or a combination of VCM with CFPM.

In this study, PIPC/TAZ was identified as a significant risk factor of AKI in multivariate analysis. Erdman and colleagues reported that PIPC/TAZ was an independent risk factor of AKI in neurocritical care patients receiving continuous infusion of hypertonic saline [15]. Our results also showed that PIPC/TAZ increases the risk of AKI. CKD and diabetes were also identified as risk factors of AKI in multivariate analysis. Karino and colleagues reported that renal dysfunction (creatinine clearance < 40 mL/min) was a risk factor of AKI in late-elderly patients who received PIPC/TAZ [3], being in agreement with our results. Meanwhile, diabetes was also identified as a risk factor of aminoglycoside-associated nephrotoxicity in the intensive care unit patients [16]. However, Diabetes was reported as the risk factor of CKD [17] as well as AKI [18]. Therefore, diabetes may be related to CKD in our study population.

Several limitations of this study should be acknowledged. First, our study was a single center, retrospective analysis. Therefore, the results might not be generalizable to other settings. Second, most of the patients in our study were elderly patients with a mean age of approximately 70 years. Therefore, approximately forty percent of the patients had eGFR < 60 mL/min/1.73m^2^. Kheterpal and colleagues reported that mild or moderate renal insufficiency has been defined as one of the AKI indexes in general surgery [19]. The median eGFR in our study population was close to the defined AKI index, and CKD was identified a risk factor of AKI in our statistical analysis. However, PIPC/TAZ has been recommended in the guidelines of nursing and healthcare-associated pneumonia [20], complicated urinary tract infection [21] and binary tract infection [22], and most of the patients apply these guidelines are elderly. There was no significant difference in eGFR between the two groups, and patients’ age was not identified as a risk factor of AKI in our statistical analysis. Moreover, PIPC/TAZ has been identified as an independent risk factor of AKI in multivariate analysis. However, there might be a statistical error in this analysis because of logistic regression analysis with small sample size. Third, we were not able to evaluate the severity by using a scoring system such as Acute Physiology and Chronic Health Evaluation (APACHE) or Sequential Organ Failure Assessment (SOFA) to predict the prognosis. Therefore, we could not rule out the possibility of AKI being caused by infectious diseases. However, 30-days mortality was not significantly different in both groups. Finally, the proportion of patients in whom a contrast media was used was significantly larger in the PIPC/TAZ group than in the CFPM group. Nevertheless, only one patient occurred AKI in the PIPC/TAZ group, and the proportion of patients using contrast media was not significantly different between the AKI group and the non-AKI group. Thus, we consider that the use of contrast media did not contribute to our results.

The mechanism of nephrotoxicity induced by PIPC/TAZ remains to be elucidated. The results of this study suggest that physicians and clinical pharmacists need to closely monitor renal function in patients receiving PIPC/TAZ.

## Conclusions

In summary, we revealed that the incidence of AKI in patients who received PIPC/TAZ monotherapy was higher than that in patients who received CFPM. Furthermore, PIPC/TAZ was shown to be independently associated with an increased risk of AKI. AKI severity was mostly mild in our study population, being similar to that in other reports. Moreover, the onset of AKI in the PIPC/TAZ group was earlier than that in the CFPM group. Therefore, we recommend that clinicians monitor renal function in patients receiving PIPC/TAZ therapy. Finally, further study is required to investigate the mechanism of nephrotoxicity associated with PIPC/TAZ.

## Data Availability

Not applicable.

## Abbreviations

AKI: acute kidney injury
CFPM: cefepime
PIPC/TAZ: piperacillin/tazobactam
SCr: serum creatinine
